# Supplementary material for: Network Analysis and Transcriptome Profiling Identify Autophagic and Mitochondrial Dysfunctions in SARS-CoV-2 Infection
Source: bioRxiv. 2020 Jul 20:2020.05.13.092536. Originally published 2020 May 14. Preprint. [Version 2] doi: 10.1101/2020.05.13.092536 (PMC7241104; doi:10.1101/2020.05.13.092536)
Supplement: Supplement 1 — Supp Fig S1. (A) Volcano plot showing DE genes that were up (red color dots) and down regulated (blue color dots) in hACE2 transduced A549 cells infected with SARS-CoV-2 (high MOI). (B) Top 25 pathways from the pathway enrichment analysis of the DE genes from the mock vs. SARS-CoV-2 (high MOI) comparison is presented as a horizontal bar plot, where x axis represents the -log10 transformed q value and the color of the horizontal bar is scaled blue to red representing low to high q values, respectively. (C) Volcano plot showing DE genes that were up (red color dots) and down regulated (blue color dots) in SARS-CoV-2 (low MOI) infected A549 cells that were transduced with hACE2. (D) Top 25 pathways from the pathway enrichment analysis of the DE genes from the mock vs. SARS-CoV-2 (low MOI) comparison is presented as a horizontal bar plot, where x axis represents the -log10 transformed q value and the color of the horizontal bar is scaled blue to red representing low to high q values, respectively. DE: differentially expressed; MOI: multiplicity of infection. (E) Plot showing correlation between marker genes from different lung subpopulations (on x-axis) and hACE2 transduced A549 and Calu3 cells lines (color coded independent samples with legend at the bottom of the plot). Supp Fig S2. (A) Correlation plot between mean gene expression from SARS-CoV-2 infected hACE2 transduced A549 (x axis) and Calu3 (y axis) cells. (B) Venn diagram showing overlap between DE genes from mock vs. SARS-CoV-2 A549 and Calu3 cell comparisons. (C) Pathway enrichment summary map for mock vs. SARS-CoV-2 comparisons in Calu3 (blue nodes) and hACE2 transduced A549 (red nodes) cells. Each node represents a pathway/biological process (BP). The node size is proportional to the number of DE genes overlapping with the BP. The nodes that share genes are connected with edges. The black circle outlines group the gene ontology (GO) terms of similar BPs. Single color nodes are pathways that are distinctly e [file media-1.pdf]

Supp Figure S1

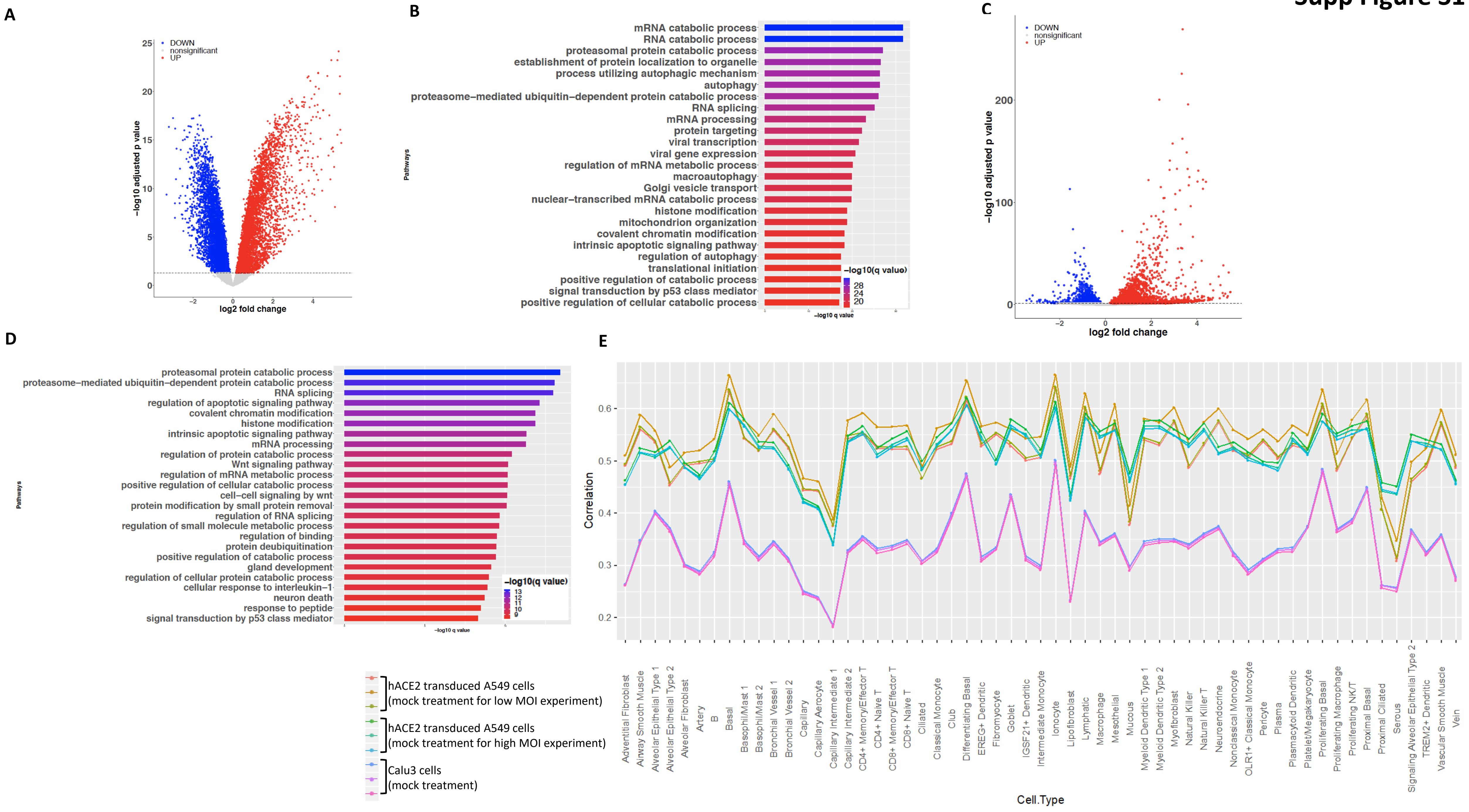

Supp Figure S2

A

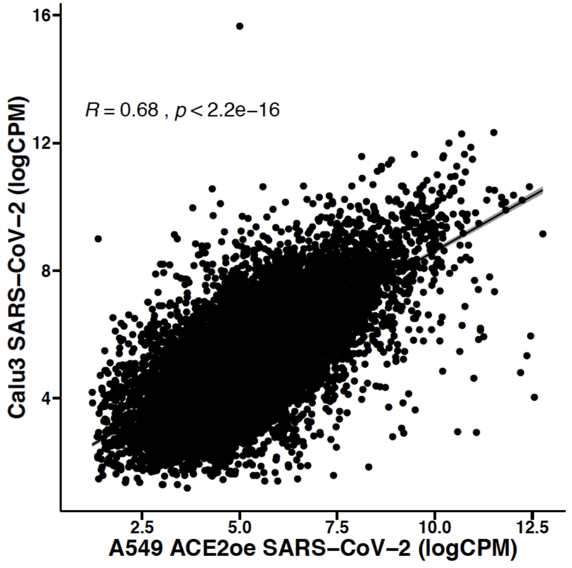

B

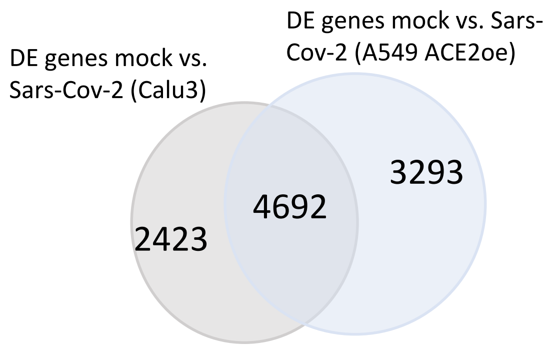

C

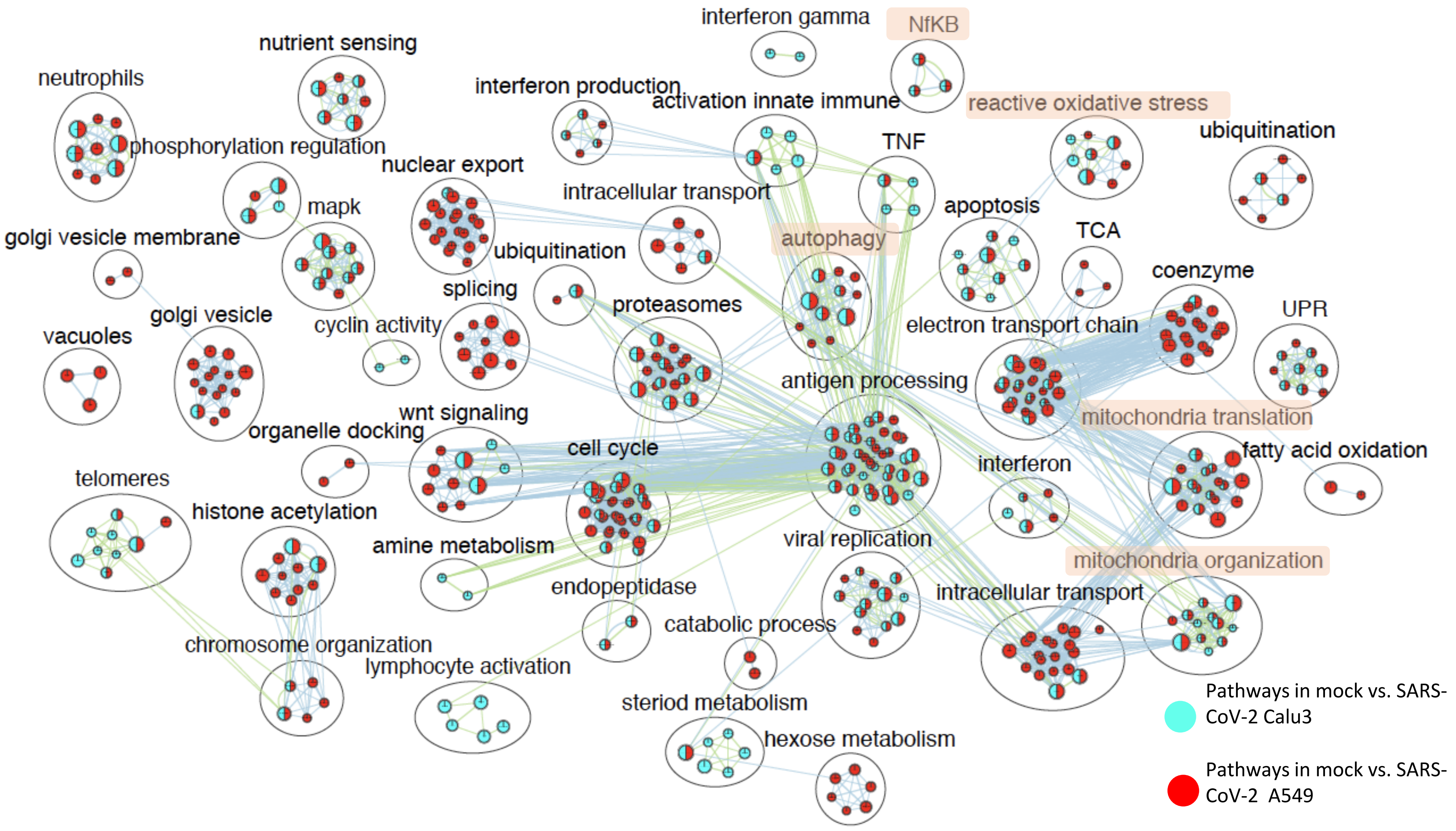

Supp Figure S3

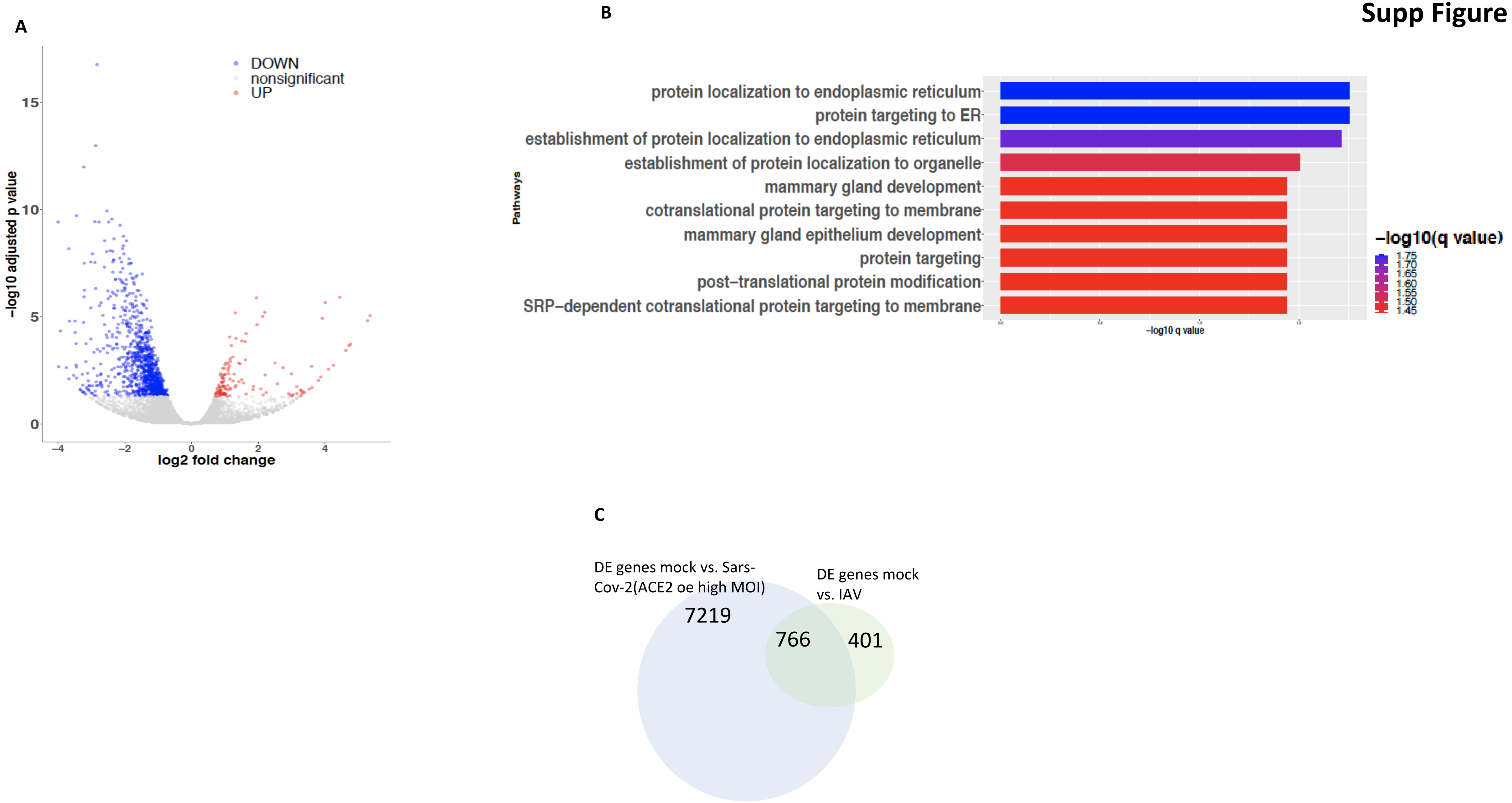

Supp Figure S4

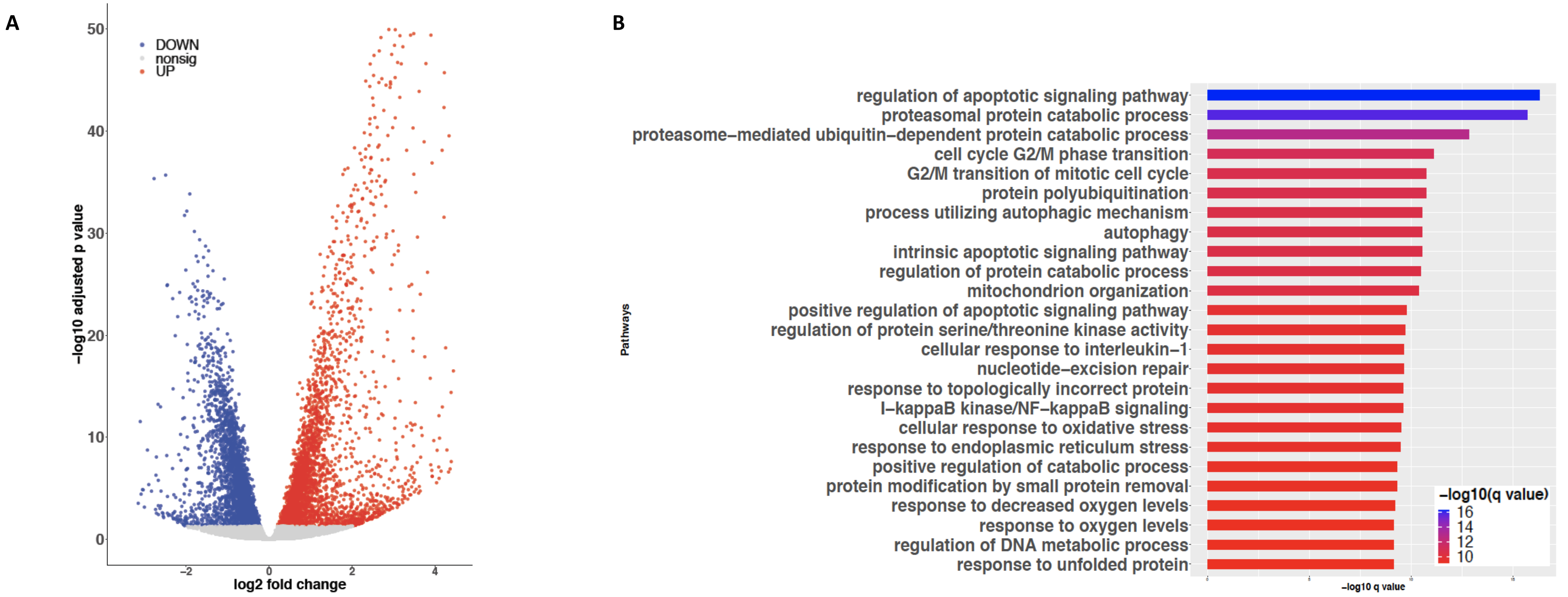

Supp Figure S5

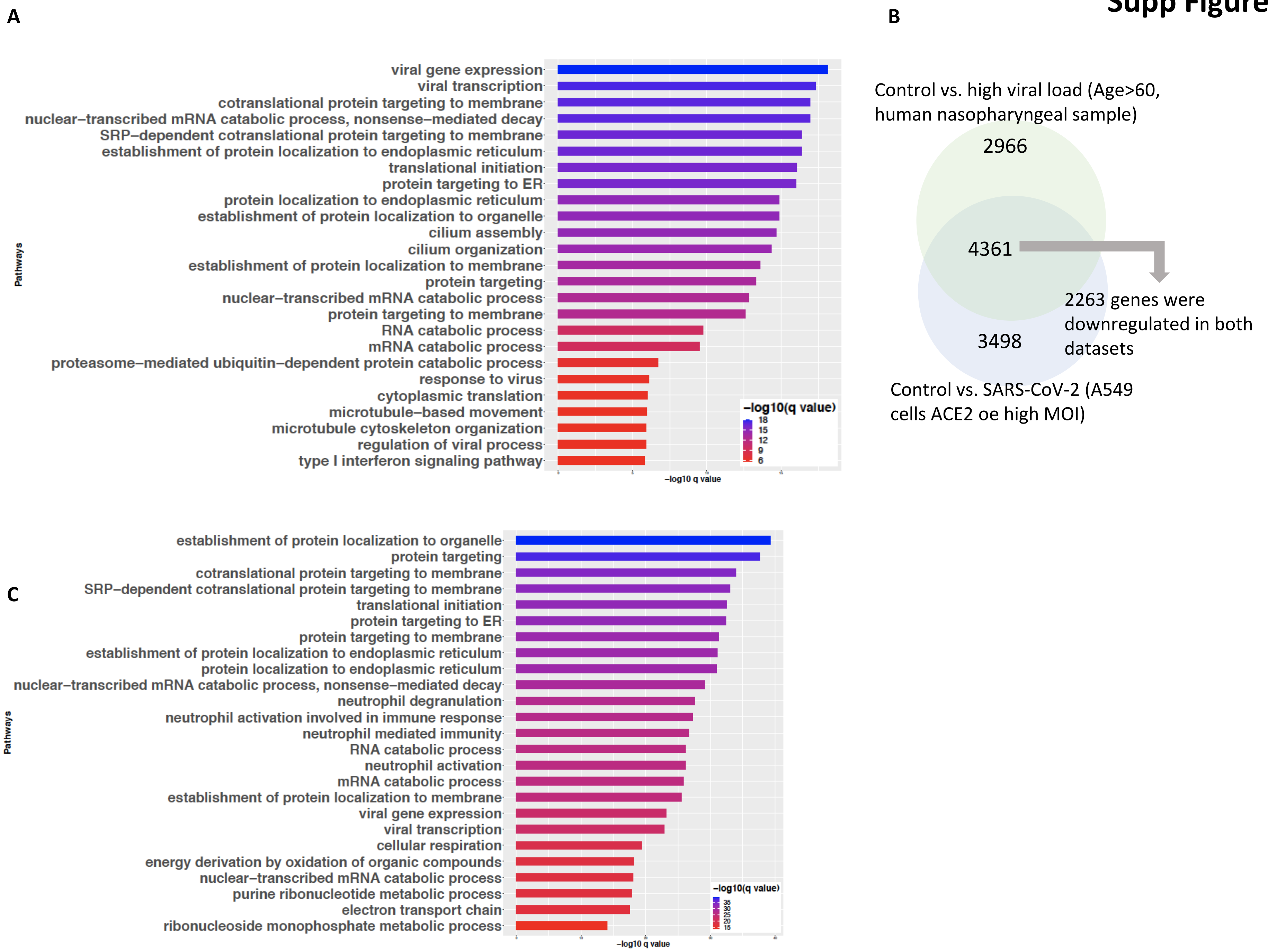

Supp Figure S6

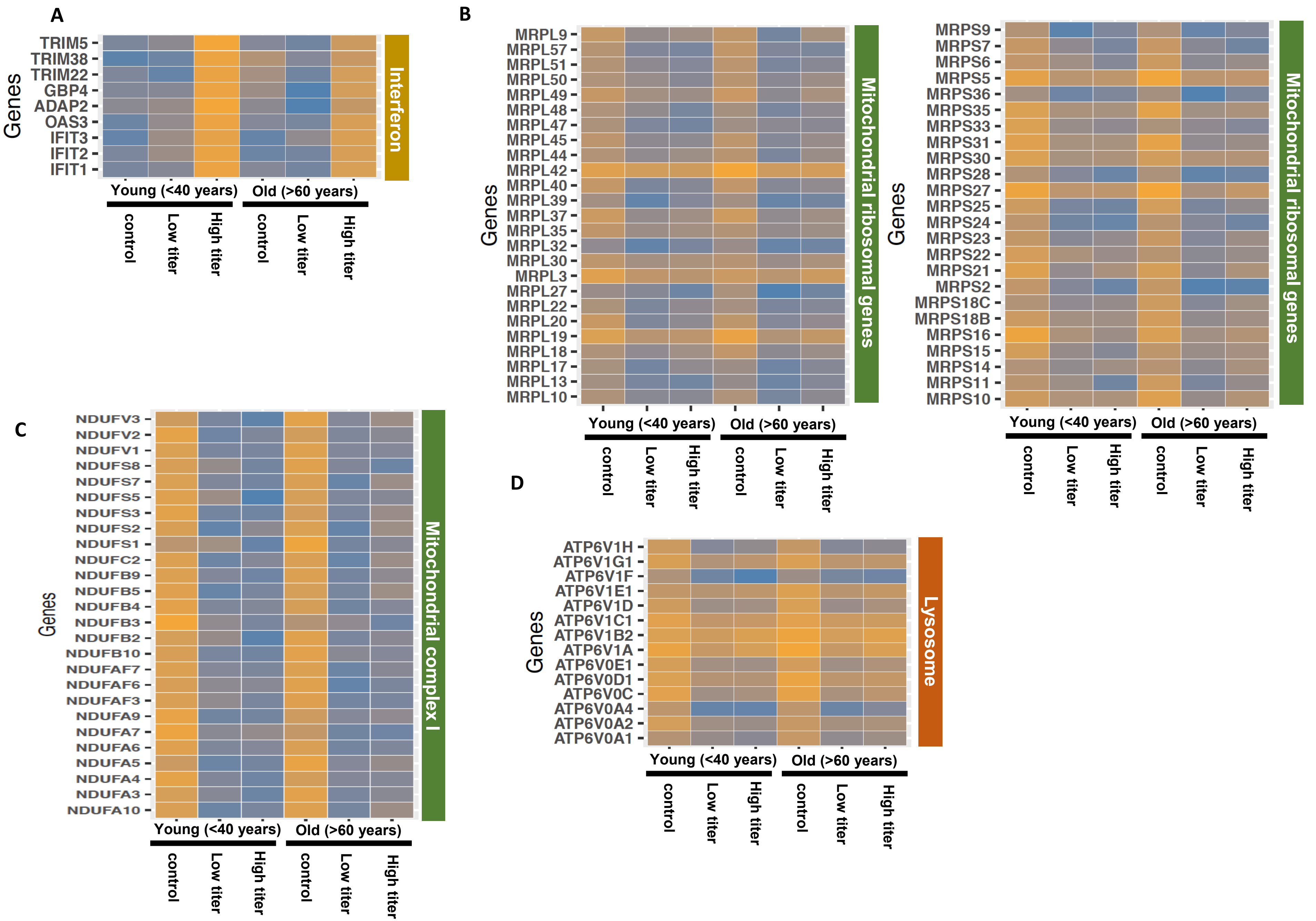

Supp Figure S7

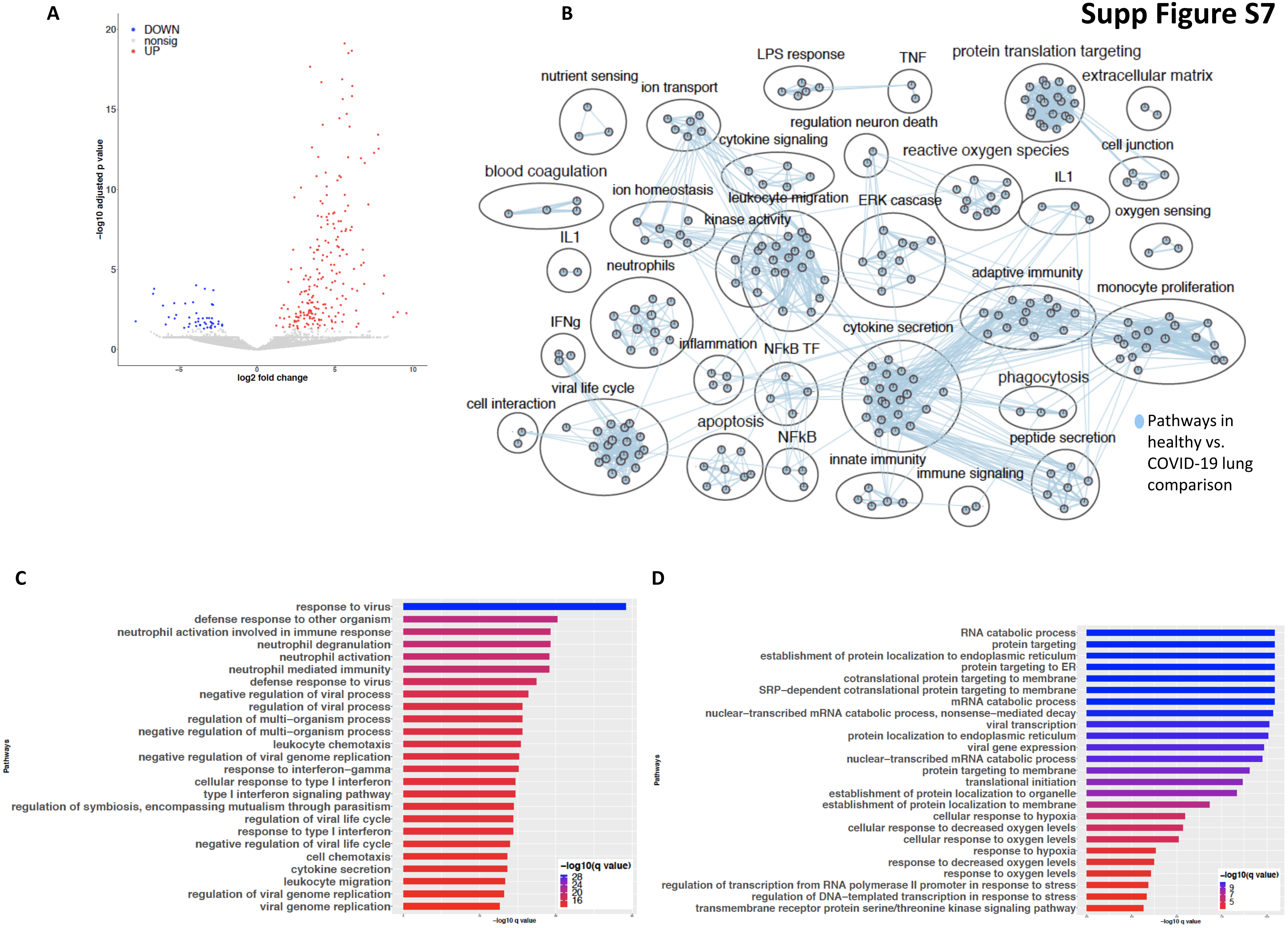

Table S1: Consensus module name and					
Module Name	Module Size	Significant Genes Overlap	Module Name	Module Size	Significant Genes Overlap
bisque4	51	27	magenta	158	77
black	176	118	mediumpurp	56	28
blue	2093	1806	midnightblue	123	78
brown	431	308	orange	83	42
brown4	52	36	orangered4	58	36
cyan	125	92	paleturquois	77	60
darkgreen	89	55	pink	162	128
darkgrey	84	55	plum1	62	43
darkmagenta	64	54	purple	144	74
darkolivegre	66	46	red	183	116
darkorange	81	44	royalblue	106	54
darkorange2	53	27	saddlebrown	78	36
darkred	94	67	salmon	127	68
darkturquois	86	43	sienna3	63	32
floralwhite	53	36	skyblue	78	48
green	186	87	skyblue3	62	42
greenyellow	139	101	steelblue	78	31
grey60	113	70	tan	139	89
ivory	53	39	turquoise	2096	1804
lightcyan	114	79	violet	69	36
lightcyan1	53	29	white	79	47
lightgreen	108	59	yellow	314	171
lightsteelblu	55	38	yellowgreen	62	43
lightyellow	107	66			

Table S2: Marker genes in each cluster						
gene	cluster	p_val	avg_logFC	pct.1	pct.2	p_val_adj
LUM	Adventitial Fibroblast	0	2.786590029	0.996	0.046	0
C1R	Adventitial Fibroblast	0	2.230799138	0.957	0.104	0
C1S	Adventitial Fibroblast	0	2.12550214	0.964	0.067	0
COL6A2	Adventitial Fibroblast	0	1.949707642	0.969	0.095	0
C3	Adventitial Fibroblast	0	1.625204903	0.806	0.095	0
EFEMP1	Adventitial Fibroblast	0	1.579792258	0.763	0.108	0
MYC	Adventitial Fibroblast	0	1.533787556	0.826	0.13	0
RARRES1	Adventitial Fibroblast	0	1.387014784	0.601	0.064	0
LTBP4	Adventitial Fibroblast	0	1.34734777	0.903	0.094	0
SERPINA3	Adventitial Fibroblast	0	1.321580056	0.637	0.027	0
MEG3	Adventitial Fibroblast	0	1.266829057	0.596	0.026	0
FSTL1	Adventitial Fibroblast	0	1.239723918	0.749	0.075	0
PLTP	Adventitial Fibroblast	0	1.16386539	0.801	0.079	0
UAP1	Adventitial Fibroblast	0	1.077900945	0.614	0.12	0
C11orf96	Adventitial Fibroblast	1.50E-291	1.114677374	0.598	0.112	3.06E-287
FHL1	Adventitial Fibroblast	1.30E-281	1.117944403	0.862	0.29	2.65E-277
CCL2	Adventitial Fibroblast	2.11E-279	2.330734285	0.668	0.156	4.30E-275
IGFBP7	Adventitial Fibroblast	7.64E-207	1.17851979	0.878	0.334	1.56E-202
JUNB	Adventitial Fibroblast	6.55E-196	1.554021352	0.955	0.75	1.33E-191
SOCS3	Adventitial Fibroblast	3.69E-170	1.268265343	0.811	0.375	7.51E-166
DUSP1	Adventitial Fibroblast	7.44E-166	1.093371235	0.982	0.866	1.51E-161
MT1A	Adventitial Fibroblast	8.50E-155	1.263691019	0.506	0.133	1.73E-150
FOSB	Adventitial Fibroblast	1.22E-152	1.320675502	0.695	0.28	2.49E-148
PNRC1	Adventitial Fibroblast	1.45E-122	1.003578227	0.883	0.641	2.95E-118
IL6	Adventitial Fibroblast	6.38E-110	1.625276577	0.452	0.142	1.30E-105
GADD45B	Adventitial Fibroblast	5.58E-95	1.17446996	0.697	0.365	1.14E-90
ACTA2	Airway Smooth Muscle	0	3.46383406	0.999	0.082	0
MYL9	Airway Smooth Muscle	0	3.017381895	0.999	0.179	0
IGFBP7	Airway Smooth Muscle	0	2.321618285	0.999	0.331	0
TPM1	Airway Smooth Muscle	0	1.741045717	0.981	0.273	0
MFGE8	Airway Smooth Muscle	0	1.382008804	0.876	0.108	0
C11orf96	Airway Smooth Muscle	0	1.35900598	0.724	0.11	0
MAP1B	Airway Smooth Muscle	0	1.045935725	0.727	0.04	0
MT1A	Airway Smooth Muscle	2.42E-110	1.028382373	0.418	0.133	4.92E-106
CD55	Alveolar Epithelial Type	0	1.765846081	0.964	0.374	0
ANXA3	Alveolar Epithelial Type	0	1.544484009	0.919	0.158	0
CD9	Alveolar Epithelial Type	0	1.426255262	0.996	0.661	0
SLC39A8	Alveolar Epithelial Type	0	1.409703442	0.923	0.115	0
MYL9	Alveolar Epithelial Type	0	1.39117199	0.956	0.176	0
RAB11FIP1	Alveolar Epithelial Type	0	1.375543319	0.899	0.189	0
CLDN4	Alveolar Epithelial Type	0	1.292853547	0.817	0.107	0
KLF6	Alveolar Epithelial Type	0	1.241313753	0.961	0.629	0
ICAM1	Alveolar Epithelial Type	0	1.20581191	0.909	0.275	0

CAV2	Alveolar Epithelial Type	0	1.191728797	0.952	0.275	0
APLP2	Alveolar Epithelial Type	0	1.136831369	0.948	0.492	0
SLPI	Alveolar Epithelial Type	0	1.878738268	0.996	0.176	0
CXCL2	Alveolar Epithelial Type	0	1.723323158	0.839	0.225	0
LAMP3	Alveolar Epithelial Type	0	1.329693634	0.92	0.02	0
C11orf96	Alveolar Epithelial Type	0	1.222678159	0.793	0.074	0
ABCA3	Alveolar Epithelial Type	0	1.16127674	0.879	0.025	0
AREG	Alveolar Epithelial Type	0	1.103382842	0.721	0.184	0
SDC4	Alveolar Epithelial Type	0	1.070143991	0.871	0.178	0
SDR16C5	Alveolar Epithelial Type	0	1.036025167	0.813	0.038	0
LUM	Alveolar Fibroblast	0	3.078860561	0.987	0.035	0
COL6A2	Alveolar Fibroblast	0	1.538556201	0.91	0.086	0
FHL1	Alveolar Fibroblast	0	1.523997458	0.94	0.281	0
C1S	Alveolar Fibroblast	0	1.500879306	0.856	0.059	0
C1R	Alveolar Fibroblast	0	1.288662061	0.819	0.097	0
MACF1	Alveolar Fibroblast	0	1.241721333	0.803	0.162	0
G0S2	Alveolar Fibroblast	0	1.21200183	0.602	0.07	0
LTBP4	Alveolar Fibroblast	0	1.146861184	0.82	0.086	0
LIMCH1	Alveolar Fibroblast	0	1.099595346	0.785	0.096	0
DKK3	Alveolar Fibroblast	0	1.075098057	0.765	0.049	0
SERPINA3	Alveolar Fibroblast	0	1.036220536	0.43	0.024	0
CCL2	Alveolar Fibroblast	4.21E-165	1.352282783	0.424	0.155	8.57E-161
MT1A	Artery	0	1.911629805	0.654	0.124	0
CXCL2	Artery	0	1.640122089	0.695	0.25	0
EMP1	Artery	0	1.385755577	0.794	0.232	0
CTNNAL1	Artery	0	1.359097047	0.861	0.135	0
EPAS1	Artery	0	1.295336923	0.99	0.344	0
MT2A	Artery	0	1.250994712	0.993	0.758	0
TFPI	Artery	0	1.225280101	0.901	0.244	0
TSPAN7	Artery	0	1.083194188	0.796	0.118	0
IL33	Artery	0	1.040389236	0.767	0.078	0
SRPX	Artery	0	1.003528294	0.737	0.061	0
IL6	Artery	3.11E-256	1.272206529	0.439	0.137	6.33E-252
LTB	B	0	1.703832645	0.913	0.098	0
RPS27	B	0	1.377221664	1	0.995	0
RPL39	B	0	1.138428334	1	0.979	0
RPL13A	B	0	1.12874843	1	0.995	0
RPS23	B	0	1.081575606	1	0.986	0
RPS8	B	0	1.065513987	1	0.984	0
RPL23A	B	0	1.038917475	1	0.983	0
RPSA	B	0	1.025482632	1	0.906	0
RPL37	B	0	1.024511068	1	0.973	0
RPS18	B	0	1.015328931	1	0.993	0
RCSD1	B	0	1.01495427	0.799	0.133	0
RPL34	B	0	1.001440921	1	0.992	0

KRT15	Basal	0	1.884304498	0.784	0.02	0
ERRFI1	Basal	0	1.167132207	0.78	0.139	0
LAMB3	Basal	0	1.110547199	0.837	0.048	0
MYC	Basal	2.98E-290	1.05906622	0.697	0.132	6.07E-286
IER3	Basal	8.88E-254	1.260804702	0.729	0.165	1.81E-249
CD9	Basal	8.60E-170	1.053711092	0.97	0.664	1.75E-165
CXCL1	Basal	3.14E-169	1.954447191	0.537	0.121	6.39E-165
TSC22D3	Basophil/Mast 1	0	1.038745298	0.973	0.656	0
AREG	Basophil/Mast 1	2.18E-228	1.245803697	0.553	0.208	4.43E-224
BIRC3	Basophil/Mast 2	0	1.799216945	0.924	0.218	0
SRGN	Basophil/Mast 2	0	1.795134864	1	0.792	0
AREG	Basophil/Mast 2	0	1.755138551	0.815	0.21	0
NFKBIA	Basophil/Mast 2	0	1.701406796	1	0.771	0
CREM	Basophil/Mast 2	0	1.505479187	0.88	0.189	0
CPM	Basophil/Mast 2	0	1.253602618	0.904	0.212	0
CSF1	Basophil/Mast 2	0	1.174825666	0.799	0.131	0
TNFAIP3	Basophil/Mast 2	0	1.140770708	0.931	0.25	0
TNFRSF9	Basophil/Mast 2	0	1.085641938	0.73	0.01	0
PTGS2	Basophil/Mast 2	0	1.02255169	0.609	0.057	0
FOSB	Basophil/Mast 2	1.08E-306	1.07249491	0.897	0.278	2.19E-302
GADD45B	Basophil/Mast 2	2.20E-251	1.215040961	0.929	0.363	4.49E-247
SDCBP	Basophil/Mast 2	1.05E-237	1.061626093	0.978	0.639	2.13E-233
DDIT4	Basophil/Mast 2	3.41E-237	1.026575298	0.895	0.328	6.94E-233
PLIN2	Basophil/Mast 2	2.34E-198	1.108371384	0.857	0.348	4.77E-194
SELE	Bronchial Vessel 1	0	1.84341532	0.343	0.01	0
MYC	Bronchial Vessel 1	0	1.25981007	0.719	0.132	0
IGFBP7	Bronchial Vessel 1	3.15E-290	1.726165669	0.965	0.334	6.41E-286
EMP1	Bronchial Vessel 1	1.72E-205	1.165592937	0.802	0.241	3.51E-201
SOCS3	Bronchial Vessel 1	3.26E-188	1.384362615	0.879	0.375	6.64E-184
IL6	Bronchial Vessel 1	1.12E-91	1.775003887	0.455	0.142	2.28E-87
CCL2	Bronchial Vessel 1	6.37E-47	1.071184335	0.398	0.159	1.30E-42
EMP1	Bronchial Vessel 2	1.57E-183	1.706785826	0.928	0.242	3.21E-179
SOCS3	Bronchial Vessel 2	9.15E-132	1.636335778	0.945	0.376	1.86E-127
MT2A	Bronchial Vessel 2	4.07E-75	1.465991051	0.991	0.763	8.28E-71
SOCS2	Bronchial Vessel 2	1.43E-71	1.010244019	0.57	0.167	2.92E-67
CCL2	Bronchial Vessel 2	2.07E-41	1.262389577	0.468	0.16	4.21E-37
EPAS1	Capillary	0	1.569223972	0.987	0.279	0
AKAP12	Capillary	0	1.235216151	0.575	0.097	0
MT2A	Capillary	0	1.192902856	0.996	0.734	0
CD59	Capillary	0	1.006696798	0.977	0.619	0
HLA-E	Capillary	0	1.001252428	1	0.873	0
HLA-E	Capillary Aerocyte	0	1.630291389	1	0.88	0
APP	Capillary Aerocyte	0	1.307051117	0.919	0.252	0
SERPINE1	Capillary Aerocyte	0	1.206177658	0.57	0.078	0
PDLIM1	Capillary Aerocyte	0	1.201891905	0.976	0.596	0

ESAM	Capillary Aerocyte	0	1.15382022	0.918	0.203	0
RHOB	Capillary Aerocyte	0	1.148835301	0.93	0.468	0
IFNGR1	Capillary Aerocyte	0	1.104567853	0.906	0.404	0
CAV2	Capillary Aerocyte	0	1.052730529	0.899	0.242	0
JUN	Capillary Aerocyte	0	1.00881753	0.979	0.593	0
EPAS1	Capillary Intermediate 1	0	1.456091416	1	0.353	0
SERPINE1	Capillary Intermediate 1	0	1.371532474	0.798	0.104	0
CX3CL1	Capillary Intermediate 1	0	1.318659672	0.887	0.135	0
HLA-E	Capillary Intermediate 1	0	1.274783513	1	0.886	0
AKAP12	Capillary Intermediate 1	0	1.256393851	0.838	0.144	0
ESAM	Capillary Intermediate 1	0	1.224291981	0.992	0.243	0
PDLIM1	Capillary Intermediate 1	0	1.156240857	0.995	0.618	0
APP	Capillary Intermediate 1	0	1.056021484	0.978	0.29	0
ARHGAP29	Capillary Intermediate 1	0	1.008913591	0.944	0.216	0
RHOB	Capillary Intermediate 1	6.02E-285	1.085276589	0.992	0.494	1.22E-280
CSF3	Capillary Intermediate 1	5.33E-284	1.082588688	0.525	0.096	1.09E-279
EMP1	Capillary Intermediate 1	1.19E-271	1.032287386	0.812	0.239	2.43E-267
ICAM1	Capillary Intermediate 1	1.56E-226	1.136090767	0.803	0.279	3.17E-222
MT2A	Capillary Intermediate 1	3.61E-196	1.0293594	1	0.761	7.35E-192
EPAS1	Capillary Intermediate 2	2.92E-239	1.260948699	0.963	0.354	5.95E-235
IL32	CD4+ Memory/Effector T	0	1.367355189	0.958	0.353	0
LTB	CD4+ Memory/Effector T	0	1.208570503	0.657	0.08	0
BTG1	CD4+ Memory/Effector T	0	1.12216113	0.992	0.707	0
TSC22D3	CD4+ Memory/Effector T	0	1.106162868	0.967	0.648	0
CCL5	CD4+ Memory/Effector T	0	1.011757492	0.731	0.145	0
LTB	CD4+ Naive T	0	1.661789742	0.958	0.095	0
RPS27	CD4+ Naive T	0	1.517082546	1	0.995	0
RPS12	CD4+ Naive T	0	1.295506121	1	0.985	0
RPS15A	CD4+ Naive T	0	1.256920807	1	0.987	0
RPL34	CD4+ Naive T	0	1.250350415	1	0.992	0
RPL39	CD4+ Naive T	0	1.241588509	1	0.979	0
RPS6	CD4+ Naive T	0	1.234368294	1	0.987	0
RPL32	CD4+ Naive T	0	1.204709159	1	0.992	0
RPS3A	CD4+ Naive T	0	1.181221239	1	0.985	0
RPL30	CD4+ Naive T	0	1.175999212	1	0.983	0
RPL31	CD4+ Naive T	0	1.16513278	1	0.965	0
RPS18	CD4+ Naive T	0	1.148329926	1	0.993	0
RPL13A	CD4+ Naive T	0	1.139367342	1	0.995	0
RPL4	CD4+ Naive T	0	1.127375171	1	0.893	0
RPL36A	CD4+ Naive T	0	1.121459321	0.915	0.417	0
RPL37	CD4+ Naive T	0	1.111473185	1	0.973	0
GAS5	CD4+ Naive T	0	1.104400027	0.95	0.42	0
RPL3	CD4+ Naive T	0	1.103919365	1	0.992	0
EEF1G	CD4+ Naive T	0	1.100430572	1	0.942	0
RPL23A	CD4+ Naive T	0	1.089333354	1	0.983	0

RPL35A	CD4+ Naive T	0	1.086961826	1	0.985	0
RPS27A	CD4+ Naive T	0	1.082878265	1	0.995	0
RPSA	CD4+ Naive T	0	1.079851771	1	0.906	0
RPL38	CD4+ Naive T	0	1.064240747	1	0.945	0
RPS8	CD4+ Naive T	0	1.035175388	1	0.984	0
RPL11	CD4+ Naive T	0	1.023419135	1	0.993	0
RPL10A	CD4+ Naive T	0	1.017963561	1	0.961	0
RPL5	CD4+ Naive T	0	1.015173456	1	0.936	0
RPS4X	CD4+ Naive T	0	1.008160883	1	0.978	0
RPS23	CD4+ Naive T	0	1.000084552	1	0.986	0
CCL5	CD8+ Memory/Effector T	0	1.65924011	0.966	0.158	0
RPS27	CD8+ Memory/Effector T	0	1.156428402	1	0.995	0
IL32	CD8+ Memory/Effector T	0	1.027832655	0.961	0.37	0
CCL5	CD8+ Naive T	0	2.014546049	0.96	0.147	0
IL32	CD8+ Naive T	0	1.098806478	0.947	0.362	0
SAA1	Ciliated	0	2.252977849	0.446	0.057	0
SAA2	Ciliated	0	2.002001417	0.397	0.031	0
ODF3B	Ciliated	0	1.994019763	0.977	0.163	0
CCDC146	Ciliated	0	1.64055031	0.952	0.014	0
LCN2	Ciliated	0	1.638736568	0.632	0.071	0
CCDC170	Ciliated	0	1.617381413	0.951	0.021	0
DNAH5	Ciliated	0	1.483915487	0.93	0.013	0
EFHC1	Ciliated	0	1.479297433	0.96	0.077	0
LRRC23	Ciliated	0	1.435576718	0.932	0.029	0
RSPH9	Ciliated	0	1.388353707	0.902	0.007	0
CLDN4	Ciliated	0	1.326561191	0.924	0.101	0
SRI	Ciliated	0	1.313381775	0.971	0.406	0
CRNDE	Ciliated	0	1.237282143	0.912	0.08	0
NUCB2	Ciliated	0	1.216717569	0.893	0.216	0
DMKN	Ciliated	0	1.188792664	0.905	0.076	0
CDS1	Ciliated	0	1.150885811	0.886	0.032	0
AKAP9	Ciliated	0	1.149054831	0.937	0.267	0
MLF1	Ciliated	0	1.114118609	0.902	0.115	0
STK33	Ciliated	0	1.100667595	0.852	0.009	0
DYNC2H1	Ciliated	0	1.078888935	0.871	0.029	0
PPIL6	Ciliated	0	1.068052926	0.842	0.012	0
CCDC113	Ciliated	0	1.041213556	0.827	0.005	0
ALCAM	Ciliated	0	1.026016414	0.89	0.139	0
S100A8	Classical Monocyte	0	3.15071556	0.981	0.261	0
S100A9	Classical Monocyte	0	3.14335825	0.994	0.426	0
CTSS	Classical Monocyte	0	1.377304718	0.998	0.519	0
CSTA	Classical Monocyte	0	1.231830039	0.943	0.263	0
LST1	Classical Monocyte	0	1.108474501	0.975	0.294	0
AP1S2	Classical Monocyte	0	1.031417561	0.825	0.218	0
RPL39	Classical Monocyte	0	1.000918679	1	0.978	0

SLPI	Club	0	2.671090349	0.987	0.213	0
CLDN4	Club	0	1.056889215	0.808	0.108	0
ATP1B1	Club	0	1.045449346	0.814	0.221	0
SOX4	Club	0	1.012977507	0.665	0.136	0
AREG	Club	1.45E-144	1.081117553	0.541	0.211	2.95E-140
SERPINB3	Differentiating Basal	0	2.835092522	0.706	0.012	0
CLDN4	Differentiating Basal	0	1.667690272	0.94	0.114	0
KRT15	Differentiating Basal	0	1.146520815	0.615	0.022	0
SERPINB4	Differentiating Basal	0	1.048640523	0.426	0.006	0
MDK	Differentiating Basal	1.69E-247	1.261617891	0.804	0.14	3.45E-243
SLPI	Differentiating Basal	2.04E-200	1.991293603	0.909	0.221	4.15E-196
RPLP0	Differentiating Basal	3.71E-143	1.170810819	1	0.916	7.56E-139
CXCL1	Differentiating Basal	2.44E-124	1.16682453	0.589	0.122	4.97E-120
EREG	EREG+ Dendritic	7.08E-235	1.016147962	0.599	0.043	1.44E-230
G0S2	EREG+ Dendritic	6.84E-87	1.705843756	0.514	0.08	1.39E-82
IER3	EREG+ Dendritic	1.20E-81	1.133486894	0.711	0.168	2.45E-77
AREG	EREG+ Dendritic	2.17E-79	1.523660327	0.803	0.214	4.41E-75
NAMPT	EREG+ Dendritic	1.16E-78	1.109432692	0.965	0.375	2.36E-74
SRGN	EREG+ Dendritic	7.32E-67	1.190812698	1	0.793	1.49E-62
IL1B	EREG+ Dendritic	9.64E-64	1.194330871	0.676	0.169	1.96E-59
PLAUR	EREG+ Dendritic	3.24E-63	1.112441964	0.859	0.297	6.61E-59
C15orf48	EREG+ Dendritic	1.34E-60	1.186129022	0.577	0.13	2.72E-56
HLA-DQA1	EREG+ Dendritic	1.49E-47	1.003512303	0.944	0.453	3.04E-43
LTBP1	Fibromyocyte	0	1.195961055	0.827	0.035	0
GEM	Fibromyocyte	6.67E-280	1.453617765	0.816	0.049	1.36E-275
DKK3	Fibromyocyte	3.68E-258	1.543171677	0.878	0.062	7.48E-254
ACTA2	Fibromyocyte	5.21E-241	3.067765367	0.99	0.09	1.06E-236
COL6A2	Fibromyocyte	2.34E-160	1.224129253	0.908	0.101	4.77E-156
MYL9	Fibromyocyte	9.99E-124	2.09425671	0.99	0.187	2.03E-119
FILIP1L	Fibromyocyte	4.14E-98	1.401994947	0.857	0.159	8.43E-94
FHL1	Fibromyocyte	3.02E-85	1.656235034	0.98	0.293	6.16E-81
IGFBP7	Fibromyocyte	1.23E-67	1.643721605	0.99	0.337	2.50E-63
TPM1	Fibromyocyte	4.02E-59	1.153090286	0.929	0.28	8.19E-55
GADD45B	Fibromyocyte	5.46E-11	1.156686377	0.633	0.367	1.11E-06
LCN2	Goblet	0	3.342277345	0.994	0.08	0
SERPINB3	Goblet	0	3.064383002	0.912	0.013	0
CFB	Goblet	0	1.365219525	0.862	0.075	0
CLDN10	Goblet	0	1.331012713	0.843	0.013	0
ASS1	Goblet	0	1.320424954	0.893	0.077	0
SERPINB4	Goblet	0	1.194991617	0.535	0.007	0
CDC42EP5	Goblet	0	1.07067372	0.855	0.041	0
CREB3L1	Goblet	0	1.011198103	0.774	0.041	0
BIK	Goblet	0	1.008297567	0.83	0.028	0
SPINT1	Goblet	2.24E-262	1.030034356	0.931	0.114	4.55E-258
MDK	Goblet	1.16E-260	2.032325437	0.981	0.141	2.36E-256

CLDN4	Goblet	9.88E-252	1.532100344	0.937	0.116	2.01E-247
SLPI	Goblet	1.87E-185	3.184910521	1	0.222	3.81E-181
C3	Goblet	4.54E-170	1.032222497	0.748	0.099	9.23E-166
CXCL1	Goblet	2.50E-157	1.491889238	0.799	0.122	5.08E-153
HEBP2	Goblet	1.34E-152	1.105661522	0.899	0.186	2.72E-148
ATP1B1	Goblet	1.13E-140	1.260680085	0.95	0.228	2.31E-136
C15orf48	Goblet	2.98E-126	1.775894637	0.736	0.13	6.07E-122
NUCB2	Goblet	1.11E-119	1.411097533	0.874	0.228	2.26E-115
XBP1	Goblet	2.53E-111	1.6625422	0.981	0.423	5.14E-107
CD9	Goblet	2.20E-66	1.07506251	0.981	0.665	4.47E-62
HLA-DQA1	IGSF21+ Dendritic	1.60E-156	1.561736414	0.979	0.452	3.26E-152
LST1	Intermediate Monocyte	3.34E-163	1.75360999	1	0.314	6.80E-159
NAMPT	Intermediate Monocyte	8.36E-100	1.110519647	0.941	0.374	1.70E-95
SAT1	Intermediate Monocyte	5.97E-98	1.293106599	1	0.847	1.22E-93
CTSS	Intermediate Monocyte	1.03E-88	1.109284975	1	0.533	2.10E-84
SCG2	Ionocyte	0	1.547379095	0.682	0.002	0
FAM24B	Ionocyte	4.18E-104	1.166699655	0.636	0.019	8.50E-100
BIK	Ionocyte	1.04E-83	1.100839019	0.727	0.03	2.12E-79
PFN2	Ionocyte	1.54E-57	1.058895427	0.864	0.062	3.13E-53
TPD52	Ionocyte	8.30E-45	1.508468881	0.955	0.108	1.69E-40
CLDN4	Ionocyte	1.05E-37	1.333097135	0.955	0.118	2.14E-33
SOX4	Ionocyte	1.70E-28	1.175933859	0.955	0.143	3.45E-24
ATP1B1	Ionocyte	1.40E-20	1.416787075	0.955	0.23	2.85E-16
SMS	Ionocyte	3.64E-16	1.022358214	0.864	0.24	7.41E-12
CD9	Ionocyte	2.47E-15	1.917322806	1	0.666	5.02E-11
APLP2	Ionocyte	1.70E-12	1.091222178	0.955	0.499	3.46E-08
FST	Lipofibroblast	0	1.953409514	0.857	0.009	0
ALDH1A3	Lipofibroblast	0	1.447865029	0.943	0.021	0
MEDAG	Lipofibroblast	0	1.388733329	0.829	0.008	0
GFPT2	Lipofibroblast	0	1.109336779	0.857	0.012	0
MLLT11	Lipofibroblast	1.04E-303	1.296283525	0.771	0.015	2.12E-299
TFPI2	Lipofibroblast	1.23E-132	1.736232812	0.943	0.051	2.50E-128
C1S	Lipofibroblast	1.22E-102	2.259195612	0.971	0.074	2.49E-98
RARRES1	Lipofibroblast	3.83E-99	1.75237774	0.943	0.068	7.79E-95
GEM	Lipofibroblast	7.46E-89	1.220392749	0.771	0.049	1.52E-84
C3	Lipofibroblast	1.75E-84	2.484340643	1	0.1	3.55E-80
SRPX	Lipofibroblast	3.17E-84	1.568588364	0.914	0.076	6.46E-80
COL6A2	Lipofibroblast	9.79E-76	2.104969852	0.971	0.102	1.99E-71
CYP1B1	Lipofibroblast	1.06E-71	1.639201113	0.771	0.063	2.15E-67
C1R	Lipofibroblast	6.54E-71	2.074512958	0.971	0.11	1.33E-66
EFEMP1	Lipofibroblast	1.59E-65	1.35614908	0.971	0.113	3.24E-61
MYC	Lipofibroblast	1.02E-55	2.200312507	0.943	0.136	2.08E-51
LUM	Lipofibroblast	2.02E-54	1.185253536	0.657	0.054	4.11E-50
ELL2	Lipofibroblast	1.05E-47	1.032427079	0.829	0.115	2.13E-43
CREM	Lipofibroblast	6.15E-43	1.861637551	0.971	0.195	1.25E-38

MT1A	Lipofibroblast	1.06E-40	1.618671558	0.886	0.136	2.15E-36
C11orf96	Lipofibroblast	2.42E-39	1.888095111	0.771	0.116	4.93E-35
UAP1	Lipofibroblast	8.36E-37	1.034420083	0.771	0.124	1.70E-32
PHLDA1	Lipofibroblast	1.84E-36	2.030816244	0.971	0.239	3.75E-32
CCL2	Lipofibroblast	7.29E-29	1.10487582	0.829	0.16	1.48E-24
NAMPT	Lipofibroblast	4.14E-27	1.452543835	1	0.376	8.43E-23
MT2A	Lipofibroblast	2.29E-24	2.325726527	1	0.763	4.65E-20
B4GALT1	Lipofibroblast	1.14E-23	1.155291092	0.886	0.238	2.33E-19
CDKN1A	Lipofibroblast	4.60E-23	1.06836998	0.8	0.205	9.35E-19
JUNB	Lipofibroblast	1.61E-21	1.734778428	1	0.752	3.28E-17
MCL1	Lipofibroblast	2.78E-21	1.480060197	1	0.655	5.66E-17
ZFP36L1	Lipofibroblast	3.05E-21	1.463558667	0.943	0.391	6.21E-17
PNRC1	Lipofibroblast	1.47E-20	1.467478275	1	0.643	2.99E-16
SOCS3	Lipofibroblast	7.26E-20	1.23767289	0.943	0.378	1.48E-15
CEBPB	Lipofibroblast	7.75E-18	1.389968049	1	0.704	1.58E-13
DUSP1	Lipofibroblast	1.14E-15	1.224461088	1	0.867	2.32E-11
CXCL2	Lipofibroblast	1.29E-14	1.494818885	0.771	0.26	2.63E-10
IGFBP7	Lymphatic	0	2.380256735	1	0.333	0
TFPI	Lymphatic	0	1.487724254	0.961	0.253	0
AKAP12	Lymphatic	0	1.462761825	0.85	0.146	0
EFEMP1	Lymphatic	0	1.268983583	0.694	0.109	0
PPFIBP1	Lymphatic	0	1.159310744	0.872	0.144	0
THBD	Lymphatic	6.79E-242	1.126838808	0.835	0.251	1.38E-237
CD9	Lymphatic	2.99E-203	1.234843127	0.983	0.664	6.08E-199
CD59	Lymphatic	7.44E-195	1.028783841	0.991	0.657	1.51E-190
MARCO	Macrophage	0	2.201537743	0.97	0.078	0
CCL20	Macrophage	0	2.029166059	0.385	0.117	0
FTH1	Macrophage	0	1.963256098	1	0.999	0
CD68	Macrophage	0	1.809531415	0.979	0.142	0
IL1B	Macrophage	0	1.715364915	0.52	0.068	0
S100A11	Macrophage	0	1.691016679	1	0.892	0
LGALS3	Macrophage	0	1.677982675	0.997	0.608	0
ALOX5AP	Macrophage	0	1.520971685	0.983	0.356	0
CTSC	Macrophage	0	1.394627856	0.953	0.274	0
OLR1	Macrophage	0	1.359594282	0.939	0.038	0
TREM1	Macrophage	0	1.357226106	0.91	0.062	0
HLA-DQA1	Macrophage	0	1.299102429	0.969	0.306	0
CXCL5	Macrophage	0	1.287616358	0.411	0.016	0
SNX10	Macrophage	0	1.269985376	0.909	0.083	0
CXCL3	Macrophage	0	1.158748812	0.633	0.113	0
PSAP	Macrophage	0	1.147320751	0.998	0.732	0
CTSB	Macrophage	0	1.14205641	0.982	0.348	0
CTSS	Macrophage	0	1.141720974	0.993	0.402	0
LTA4H	Macrophage	0	1.132272517	0.895	0.184	0
VIM	Macrophage	0	1.066901338	1	0.837	0

CSTA	Macrophage	0	1.019709732	0.909	0.103	0
PRG4	Mesothelial	0	1.947154995	0.862	0.006	0
PAPPA	Mesothelial	0	1.211065752	0.724	0.005	0
ALDH1A3	Mesothelial	6.10E-237	1.979881986	0.897	0.021	1.24E-232
STEAP1	Mesothelial	4.50E-230	1.043967231	0.759	0.015	9.15E-226
CLDN1	Mesothelial	3.29E-119	1.246674656	0.828	0.036	6.70E-115
TFPI2	Mesothelial	1.35E-91	1.793702169	0.862	0.051	2.75E-87
C1S	Mesothelial	3.41E-83	2.019559727	0.966	0.074	6.94E-79
MEST	Mesothelial	2.50E-81	1.115955184	0.724	0.04	5.09E-77
RARRES1	Mesothelial	3.13E-81	1.932162483	0.931	0.068	6.37E-77
CFB	Mesothelial	3.14E-77	2.554189363	0.931	0.076	6.39E-73
CCDC71L	Mesothelial	3.26E-65	1.373014066	0.793	0.062	6.63E-61
C3	Mesothelial	2.63E-60	2.805293274	0.931	0.1	5.36E-56
C1R	Mesothelial	3.12E-57	1.819000063	0.966	0.11	6.36E-53
COL6A2	Mesothelial	1.74E-54	1.652506981	0.931	0.102	3.54E-50
EFEMP1	Mesothelial	4.55E-49	1.27150229	0.931	0.113	9.27E-45
CCL2	Mesothelial	2.99E-37	1.876436883	0.966	0.16	6.09E-33
ERRFI1	Mesothelial	1.05E-35	1.091746088	0.897	0.143	2.13E-31
PIM1	Mesothelial	1.79E-26	1.092363919	0.793	0.158	3.63E-22
MDK	Mesothelial	1.18E-25	1.102407455	0.759	0.143	2.41E-21
RPS4Y1	Mesothelial	1.07E-20	1.174701108	0.966	0.368	2.18E-16
RPS12	Mesothelial	9.03E-17	1.038334979	1	0.985	1.84E-12
MT2A	Mesothelial	8.76E-16	1.508219114	1	0.763	1.78E-11
RPL12	Mesothelial	2.47E-15	1.014091818	1	0.964	5.03E-11
GAS5	Mesothelial	4.39E-14	1.033018436	0.931	0.427	8.93E-10
SAA1	Mucous	0	3.740597151	0.904	0.059	0
LCN2	Mucous	0	3.71595707	0.986	0.075	0
SAA2	Mucous	0	3.263902439	0.866	0.032	0
SLPI	Mucous	0	3.001902828	0.996	0.218	0
SERPINA3	Mucous	0	2.725180975	0.723	0.027	0
CXCL1	Mucous	0	2.121790394	0.796	0.119	0
PI3	Mucous	0	2.112982356	0.627	0.012	0
MMP7	Mucous	0	2.039034049	0.823	0.019	0
RARRES1	Mucous	0	1.938771313	0.896	0.062	0
C3	Mucous	0	1.768759158	0.951	0.094	0
MDK	Mucous	0	1.453983303	0.955	0.137	0
CFB	Mucous	0	1.402986853	0.868	0.071	0
PDZK1IP1	Mucous	0	1.340861311	0.811	0.067	0
CRISP3	Mucous	0	1.221590871	0.418	0.001	0
NCOA7	Mucous	0	1.079051637	0.798	0.174	0
CCL20	Mucous	6.66E-221	1.471254462	0.684	0.173	1.36E-216
XBP1	Mucous	1.37E-188	1.05928154	0.886	0.421	2.79E-184
CCL17	Myeloid Dendritic Type 1	0	2.681912668	0.489	0.007	0
CSF2RA	Myeloid Dendritic Type 1	1.01E-238	1.446571977	0.908	0.101	2.06E-234
SERPINB9	Myeloid Dendritic Type 1	4.79E-137	1.251191502	0.771	0.116	9.74E-133

RGS10	Myeloid Dendritic Type 1	1.05E-99	1.103478553	0.916	0.236	2.14E-95
HLA-DQA1	Myeloid Dendritic Type 1	4.01E-89	1.891895134	0.992	0.453	8.16E-85
C15orf48	Myeloid Dendritic Type 1	5.97E-75	2.174083729	0.626	0.13	1.22E-70
G0S2	Myeloid Dendritic Type 1	4.33E-60	2.267628586	0.45	0.08	8.81E-56
BIRC3	Myeloid Dendritic Type 1	1.08E-32	1.308175361	0.618	0.224	2.20E-28
AREG	Myeloid Dendritic Type 1	2.37E-19	1.66027885	0.473	0.215	4.83E-15
CCL17	Myeloid Dendritic Type 2	0	2.0634761	0.256	0.007	0
HLA-DQA1	Myeloid Dendritic Type 2	3.57E-164	1.762493144	0.989	0.452	7.27E-160
AREG	Myeloid Dendritic Type 2	3.47E-39	1.014500968	0.523	0.214	7.07E-35
LUM	Myofibroblast	0	1.948272991	0.944	0.051	0
COL6A2	Myofibroblast	0	1.375075631	0.863	0.1	0
ACTA2	Myofibroblast	0	1.334396257	0.806	0.089	0
DKK3	Myofibroblast	0	1.26977861	0.875	0.06	0
RARRES1	Myofibroblast	0	1.269306754	0.694	0.066	0
C1S	Myofibroblast	0	1.259900004	0.847	0.071	0
GEM	Myofibroblast	0	1.169919284	0.669	0.047	0
LTBP2	Myofibroblast	0	1.002560274	0.746	0.025	0
FILIP1L	Myofibroblast	9.21E-212	1.113113693	0.823	0.158	1.87E-207
FHL1	Myofibroblast	1.26E-171	1.415063639	0.931	0.292	2.56E-167
CCL2	Myofibroblast	1.47E-16	1.356867396	0.339	0.16	2.99E-12
CCL5	Natural Killer	0	1.417388492	0.763	0.126	0
CCL5	Natural Killer T	0	1.931881874	0.994	0.168	0
KLRC2	Natural Killer T	0	1.136054204	0.693	0.029	0
BTG1	Natural Killer T	2.36E-150	1.133920057	0.997	0.719	4.80E-146
TSC22D3	Natural Killer T	4.84E-134	1.116378879	0.994	0.661	9.86E-130
SCG2	Neuroendocrine	0	2.839338022	0.909	0.002	0
SCG5	Neuroendocrine	0	2.29019825	0.909	0.003	0
NEFL	Neuroendocrine	0	1.626390742	0.364	0.001	0
TUBB2B	Neuroendocrine	0	1.151666149	0.727	0.004	0
MB	Neuroendocrine	8.24E-78	1.023829455	0.545	0.009	1.68E-73
DNAJC12	Neuroendocrine	1.22E-77	1.018068258	0.636	0.013	2.48E-73
MEG3	Neuroendocrine	5.81E-65	1.960754926	0.909	0.031	1.18E-60
BEX2	Neuroendocrine	1.66E-24	1.487052024	0.818	0.073	3.38E-20
PAM	Neuroendocrine	5.45E-09	1.060749077	0.636	0.113	0.000110998
VAMP2	Neuroendocrine	4.70E-08	1.034107247	1	0.432	0.000957604
EIF4A2	Neuroendocrine	9.98E-08	1.097580045	1	0.559	0.002031893
CIRBP	Neuroendocrine	1.34E-07	1.153357668	1	0.746	0.00273753
LST1	Nonclassical Monocyte	0	2.207635324	1	0.307	0
CTSS	Nonclassical Monocyte	0	1.374506188	1	0.529	0
NAP1L1	Nonclassical Monocyte	0	1.261563474	0.98	0.478	0
SAT1	Nonclassical Monocyte	0	1.200929686	1	0.845	0
PSAP	Nonclassical Monocyte	0	1.062619445	0.999	0.789	0
G0S2	OLR1+ Classical Monocy	0	2.217297121	0.85	0.078	0
SERPINB9	OLR1+ Classical Monocy	0	1.292378192	0.903	0.115	0
ATP13A3	OLR1+ Classical Monocy	0	1.151570772	0.894	0.1	0

C15orf48	OLR1+ Classical Monocy	5.00E-258	2.248304193	0.865	0.129	1.02E-253
IL1B	OLR1+ Classical Monocy	4.04E-237	2.085705497	0.942	0.167	8.23E-233
TNFRSF1B	OLR1+ Classical Monocy	2.52E-224	1.288720633	0.865	0.15	5.12E-220
ACSL1	OLR1+ Classical Monocy	6.18E-213	1.262036867	0.908	0.17	1.26E-208
IER3	OLR1+ Classical Monocy	7.82E-197	1.312593618	0.87	0.167	1.59E-192
GK	OLR1+ Classical Monocy	1.67E-183	1.04648259	0.787	0.141	3.40E-179
BCL2A1	OLR1+ Classical Monocy	1.47E-177	1.496522943	0.937	0.221	2.99E-173
SOD2	OLR1+ Classical Monocy	7.96E-177	2.417967238	1	0.335	1.62E-172
MARCKS	OLR1+ Classical Monocy	1.96E-161	1.417300987	0.942	0.265	3.99E-157
IFNGR2	OLR1+ Classical Monocy	6.29E-154	1.189193662	0.918	0.258	1.28E-149
NAMPT	OLR1+ Classical Monocy	7.29E-151	1.724893232	0.99	0.374	1.48E-146
TNFAIP3	OLR1+ Classical Monocy	1.18E-147	1.264031264	0.908	0.254	2.40E-143
S100A8	OLR1+ Classical Monocy	6.52E-140	1.992230888	0.918	0.282	1.33E-135
SRGN	OLR1+ Classical Monocy	2.62E-131	1.801445048	1	0.793	5.33E-127
CD44	OLR1+ Classical Monocy	5.66E-129	1.415068876	0.995	0.529	1.15E-124
UPP1	OLR1+ Classical Monocy	5.28E-127	1.009667986	0.918	0.303	1.08E-122
S100A9	OLR1+ Classical Monocy	4.21E-118	2.225300076	0.947	0.442	8.57E-114
SAT1	OLR1+ Classical Monocy	1.38E-112	1.425404055	1	0.847	2.81E-108
WTAP	OLR1+ Classical Monocy	1.32E-110	1.056550962	0.865	0.294	2.69E-106
TYMP	OLR1+ Classical Monocy	1.30E-103	1.057055136	0.966	0.426	2.65E-99
TNIP3	OLR1+ Classical Monocy	6.43E-102	1.189542507	0.488	0.084	1.31E-97
PLAUR	OLR1+ Classical Monocy	7.87E-97	1.043533931	0.879	0.297	1.60E-92
CEBPB	OLR1+ Classical Monocy	2.71E-86	1.073387628	0.99	0.703	5.51E-82
PNRC1	OLR1+ Classical Monocy	3.19E-82	1.044880784	0.976	0.642	6.49E-78
FTH1	OLR1+ Classical Monocy	2.88E-81	1.16973151	1	0.999	5.87E-77
NDUFA4L2	Pericyte	0	1.795066288	0.951	0.018	0
IGFBP7	Pericyte	0	1.691507961	0.999	0.322	0
MYL9	Pericyte	0	1.410912303	0.971	0.168	0
COL4A1	Pericyte	0	1.332323038	0.876	0.124	0
COL4A2	Pericyte	0	1.22262789	0.851	0.124	0
EFEMP1	Pericyte	0	1.120212673	0.798	0.096	0
STOM	Pericyte	0	1.056087623	0.944	0.434	0
COL6A2	Pericyte	0	1.035821438	0.831	0.084	0
PAG1	Pericyte	0	1.006654809	0.746	0.157	0
DNAJB9	Plasma	8.89E-194	1.25056027	0.898	0.175	1.81E-189
HERPUD1	Plasma	1.34E-151	2.419982595	0.989	0.379	2.72E-147
HSP90B1	Plasma	1.42E-124	1.947741479	0.984	0.514	2.89E-120
SPCS3	Plasma	1.05E-121	1.102438496	0.877	0.271	2.14E-117
SSR3	Plasma	1.80E-113	1.002058917	0.92	0.339	3.66E-109
XBP1	Plasma	1.41E-103	1.585231182	0.941	0.423	2.88E-99
BIRC3	Plasma	9.79E-103	1.164765868	0.802	0.223	1.99E-98
TSC22D3	Plasma	2.62E-71	1.359799408	0.93	0.662	5.33E-67
IRF4	Plasmacytoid Dendritic	0	1.049825978	0.759	0.01	0
IRF7	Plasmacytoid Dendritic	3.68E-216	2.072052305	0.993	0.156	7.49E-212
LTB	Plasmacytoid Dendritic	3.20E-137	1.314870555	0.759	0.105	6.51E-133

UGCG	Plasmacytoid Dendritic	8.75E-122	1.017679144	0.883	0.186	1.78E-117
CCDC50	Plasmacytoid Dendritic	3.51E-116	1.112789308	0.839	0.182	7.15E-112
PLP2	Plasmacytoid Dendritic	2.61E-99	1.157287125	0.949	0.303	5.32E-95
HERPUD1	Plasmacytoid Dendritic	1.62E-82	1.311617096	0.978	0.379	3.30E-78
AREG	Plasmacytoid Dendritic	5.03E-40	1.339158755	0.65	0.214	1.02E-35
C2orf88	Platelet/Megakaryocyte	0	1.264712452	0.625	0.008	0
KIF2A	Platelet/Megakaryocyte	3.33E-47	1.265350565	0.7	0.089	6.79E-43
PLA2G12A	Platelet/Megakaryocyte	1.18E-41	1.098241085	0.675	0.093	2.41E-37
MAX	Platelet/Megakaryocyte	8.44E-40	1.755674779	0.875	0.186	1.72E-35
CA2	Platelet/Megakaryocyte	1.65E-35	1.448175531	0.75	0.136	3.35E-31
RGS10	Platelet/Megakaryocyte	1.68E-30	2.079505257	0.85	0.237	3.42E-26
LIMS1	Platelet/Megakaryocyte	2.65E-30	1.815790956	0.85	0.234	5.39E-26
ODC1	Platelet/Megakaryocyte	3.49E-29	1.534518794	0.725	0.168	7.10E-25
MPP1	Platelet/Megakaryocyte	5.98E-28	1.538108224	0.725	0.168	1.22E-23
CCL5	Platelet/Megakaryocyte	1.72E-26	1.478776116	0.775	0.172	3.50E-22
TLK1	Platelet/Megakaryocyte	2.91E-25	1.016967049	0.575	0.109	5.92E-21
NCOA4	Platelet/Megakaryocyte	1.07E-21	1.974010353	0.725	0.235	2.18E-17
ACTN1	Platelet/Megakaryocyte	2.99E-18	1.086182096	0.7	0.226	6.09E-14
RNF11	Platelet/Megakaryocyte	1.18E-17	1.209985329	0.625	0.186	2.39E-13
TPM4	Platelet/Megakaryocyte	2.89E-17	1.737158971	0.85	0.452	5.89E-13
NAP1L1	Platelet/Megakaryocyte	3.40E-16	1.638362131	0.875	0.484	6.93E-12
DAB2	Platelet/Megakaryocyte	2.48E-14	1.021696239	0.625	0.217	5.05E-10
MYL12A	Platelet/Megakaryocyte	3.24E-13	1.355213784	1	0.939	6.60E-09
KRT15	Proliferating Basal	0	3.407924464	0.979	0.024	0
SERPINB3	Proliferating Basal	0	2.169756874	0.723	0.014	0
SERPINB4	Proliferating Basal	0	1.355115912	0.617	0.007	0
PHGDH	Proliferating Basal	9.43E-179	1.053567324	0.872	0.043	1.92E-174
CLDN4	Proliferating Basal	2.39E-75	1.314475006	0.957	0.117	4.87E-71
HMGA1	Proliferating Basal	4.68E-68	1.467549437	0.979	0.162	9.53E-64
MDK	Proliferating Basal	2.11E-64	1.671577677	0.915	0.142	4.29E-60
IMPDH2	Proliferating Basal	4.39E-63	1.249016769	0.936	0.16	8.93E-59
PLP2	Proliferating Basal	1.75E-39	1.153943634	0.979	0.304	3.57E-35
RPLP0	Proliferating Basal	2.32E-29	1.217851725	1	0.917	4.72E-25
EEF1G	Proliferating Basal	1.08E-26	1.029309195	1	0.943	2.20E-22
RPS4X	Proliferating Basal	1.14E-26	1.239131761	1	0.978	2.32E-22
RPL10A	Proliferating Basal	2.88E-26	1.119468718	1	0.961	5.86E-22
RPSA	Proliferating Basal	3.36E-26	1.066625617	1	0.907	6.85E-22
GAS5	Proliferating Basal	3.06E-25	1.035709113	0.957	0.427	6.22E-21
RPS18	Proliferating Basal	3.23E-25	1.094736461	1	0.993	6.58E-21
RPL3	Proliferating Basal	1.10E-23	1.051620079	1	0.992	2.24E-19
MARCO	Proliferating Macrophag	1.14E-173	1.482066785	0.996	0.276	2.33E-169
ANP32B	Proliferating Macrophag	4.03E-138	1.012402884	0.942	0.347	8.21E-134
CTSC	Proliferating Macrophag	1.16E-107	1.035996913	0.987	0.425	2.36E-103
IL1B	Proliferating Macrophag	5.19E-37	1.1376636	0.465	0.169	1.06E-32
CCL5	Proliferating NK/T	1.57E-63	1.111912828	0.8	0.171	3.20E-59

ANP32B	Proliferating NK/T	2.10E-63	1.111784203	0.905	0.348	4.28E-59
KRT15	Proximal Basal	0	3.189724773	0.962	0.022	0
SERPINB3	Proximal Basal	0	2.821699795	0.873	0.013	0
SERPINB4	Proximal Basal	0	1.446209984	0.694	0.006	0
CLDN4	Proximal Basal	6.88E-238	1.118066253	0.949	0.116	1.40E-233
MDK	Proximal Basal	9.98E-201	1.287877716	0.917	0.141	2.03E-196
SLPI	Proximal Basal	3.95E-149	1.705549977	1	0.222	8.05E-145
GAS5	Proximal Basal	2.07E-107	1.32646858	0.981	0.426	4.22E-103
RPL10A	Proximal Basal	4.53E-98	1.293657293	1	0.961	9.21E-94
RPS4X	Proximal Basal	4.58E-97	1.333222904	1	0.978	9.31E-93
RPLP0	Proximal Basal	4.86E-96	1.243300112	1	0.916	9.88E-92
RPL15	Proximal Basal	1.53E-93	1.020477184	1	0.992	3.11E-89
RPL3	Proximal Basal	6.82E-91	1.187600401	1	0.992	1.39E-86
EEF1G	Proximal Basal	1.51E-89	1.056147458	1	0.943	3.07E-85
RPL7A	Proximal Basal	3.75E-89	1.015896181	1	0.965	7.64E-85
RPS18	Proximal Basal	1.11E-87	1.145426549	1	0.993	2.26E-83
RPL12	Proximal Basal	1.06E-85	1.001940741	1	0.964	2.16E-81
RPS6	Proximal Basal	2.32E-85	1.049517565	1	0.987	4.73E-81
RPL13A	Proximal Basal	1.25E-83	1.048634389	1	0.995	2.55E-79
CD9	Proximal Basal	6.64E-80	1.099586533	1	0.665	1.35E-75
CCDC146	Proximal Ciliated	0	1.8005813	0.966	0.032	0
LRRC23	Proximal Ciliated	0	1.782314594	1	0.046	0
CCDC170	Proximal Ciliated	0	1.75272191	0.989	0.038	0
RSPH9	Proximal Ciliated	0	1.686258303	0.966	0.023	0
DNAH5	Proximal Ciliated	0	1.359674554	0.92	0.03	0
CDS1	Proximal Ciliated	0	1.21844205	0.989	0.048	0
TMEM231	Proximal Ciliated	0	1.185144322	0.955	0.025	0
PPIL6	Proximal Ciliated	0	1.167121636	0.898	0.028	0
DYNC2H1	Proximal Ciliated	0	1.121533562	0.92	0.044	0
DAW1	Proximal Ciliated	0	1.102150159	0.898	0.017	0
DZIP3	Proximal Ciliated	0	1.097537092	0.898	0.044	0
CCDC113	Proximal Ciliated	0	1.095573378	0.943	0.02	0
STK33	Proximal Ciliated	0	1.08470662	0.886	0.024	0
LRP11	Proximal Ciliated	0	1.083206231	0.898	0.039	0
RFX3	Proximal Ciliated	0	1.0370727	0.909	0.044	0
RP1	Proximal Ciliated	0	1.011050967	0.75	0.019	0
DNAL1	Proximal Ciliated	7.23E-291	1.072831619	0.932	0.056	1.47E-286
CLUAP1	Proximal Ciliated	4.32E-245	1.275572043	0.943	0.07	8.79E-241
PFN2	Proximal Ciliated	1.75E-221	1.012363236	0.852	0.061	3.56E-217
EFHC1	Proximal Ciliated	3.26E-215	1.678651304	1	0.094	6.64E-211
CRNDE	Proximal Ciliated	1.20E-204	1.581214001	0.989	0.096	2.45E-200
C11orf74	Proximal Ciliated	1.51E-200	1.100813243	0.875	0.073	3.06E-196
DMKN	Proximal Ciliated	6.56E-184	1.13442815	0.943	0.092	1.34E-179
MLF1	Proximal Ciliated	6.23E-135	1.203599814	0.943	0.13	1.27E-130
ODF3B	Proximal Ciliated	6.04E-126	1.992655448	1	0.178	1.23E-121

AZIN1	Proximal Ciliated	2.90E-116	1.00919294	0.966	0.157	5.90E-112
NUCB2	Proximal Ciliated	1.57E-93	1.789429382	0.977	0.229	3.19E-89
SLPI	Proximal Ciliated	2.83E-75	1.092712484	1	0.223	5.76E-71
SRI	Proximal Ciliated	2.14E-65	1.23820179	0.989	0.417	4.36E-61
CALM2	Proximal Ciliated	1.76E-49	1.074403239	1	0.914	3.58E-45
C6orf58	Serous	0	5.154386519	0.875	0.004	0
CRISP3	Serous	3.89E-261	1.456832454	0.458	0.004	7.93E-257
DMBT1	Serous	1.62E-140	2.845846984	1	0.038	3.30E-136
SERPINA3	Serous	3.55E-135	1.856884395	0.917	0.032	7.23E-131
LCN2	Serous	5.55E-33	1.247056433	0.75	0.082	1.13E-28
SLPI	Serous	3.05E-31	5.020015739	1	0.224	6.20E-27
NUCB2	Serous	2.48E-21	1.09353425	0.958	0.229	5.05E-17
XBP1	Serous	2.24E-15	1.320433056	1	0.424	4.56E-11
SLPI	Signaling Alveolar Epithe	0	1.752039566	0.999	0.216	0
MMP9	TREM2+ Dendritic	0	1.785258525	0.415	0.003	0
C15orf48	TREM2+ Dendritic	1.84E-190	1.694985342	0.868	0.13	3.74E-186
HLA-DQA1	TREM2+ Dendritic	1.87E-97	1.621903302	0.994	0.453	3.80E-93
CTSB	TREM2+ Dendritic	5.96E-95	1.375040707	0.994	0.489	1.21E-90
TYMP	TREM2+ Dendritic	6.22E-93	1.186156945	0.994	0.426	1.27E-88
CD68	TREM2+ Dendritic	4.61E-89	1.072619875	1	0.328	9.38E-85
CTSS	TREM2+ Dendritic	4.17E-69	1.220293139	0.975	0.533	8.48E-65
PSAP	TREM2+ Dendritic	2.39E-66	1.260881602	1	0.791	4.86E-62
FTH1	TREM2+ Dendritic	4.28E-57	1.047049261	1	0.999	8.72E-53
NUPR1	TREM2+ Dendritic	7.99E-38	1.010904675	0.698	0.342	1.63E-33
G0S2	TREM2+ Dendritic	2.17E-21	1.078858268	0.277	0.08	4.41E-17
ACTA2	Vascular Smooth Muscle	0	3.244966786	0.994	0.085	0
MYL9	Vascular Smooth Muscle	0	2.464883348	0.996	0.182	0
IGFBP7	Vascular Smooth Muscle	0	2.389330509	0.994	0.333	0
TPM1	Vascular Smooth Muscle	0	1.463259554	0.942	0.276	0
COL6A2	Vascular Smooth Muscle	0	1.367266843	0.853	0.097	0
MFGE8	Vascular Smooth Muscle	0	1.080051905	0.817	0.111	0
NTN4	Vascular Smooth Muscle	0	1.028672235	0.695	0.098	0
TGFB1I1	Vascular Smooth Muscle	0	1.009101859	0.774	0.115	0
FILIP1L	Vascular Smooth Muscle	1.96E-303	1.016281355	0.729	0.156	4.00E-299
IGFBP7	Vein	0	1.444665206	0.98	0.327	0
MT2A	Vein	0	1.421660934	0.99	0.759	0
SELE	Vein	0	1.373857296	0.264	0.007	0
MT1A	Vein	0	1.278258068	0.57	0.129	0
PLAT	Vein	0	1.167429437	0.674	0.062	0
LIFR	Vein	0	1.138642244	0.814	0.117	0
CD59	Vein	0	1.127939989	0.993	0.653	0
CYP1B1	Vein	0	1.124874509	0.545	0.055	0
EPAS1	Vein	0	1.06778236	0.979	0.348	0
RGS5	Vein	0	1.053452403	0.713	0.076	0
SRPX	Vein	0	1.038424785	0.758	0.064	0

CXCL2	Vein	5.04E-303	1.421884137	0.675	0.253	1.03E-298
CSF3	Vein	1.46E-255	1.085046807	0.39	0.095	2.96E-251
CCL2	Vein	9.19E-239	1.175490734	0.501	0.155	1.87E-234
IL6	Vein	6.08E-197	1.239169963	0.435	0.139	1.24E-192
